# Compartment and Plant Identity Shape Tree Mycobiome in a Subtropical Forest

**DOI:** 10.1128/spectrum.01347-22

**Published:** 2022-07-12

**Authors:** Hao Yang, Zhijie Yang, Quan-Cheng Wang, Yong-Long Wang, Hang-Wei Hu, Ji-Zheng He, Yong Zheng, Yusheng Yang

**Affiliations:** a Key Laboratory for Humid Subtropical Eco-geographical Processes of the Ministry of Education, Fujian Normal University, Fuzhou, China; b Sanming Forest Ecosystem National Observation and Research Station, Sanming, Fujian, China; c Faculty of Biological Science and Technology, Baotou Teacher’s College, Baotou, Inner Mongolia, China; State Key Laboratory of Mycology, Institute of Microbiology, Chinese Academy of Sciences

**Keywords:** α-diversity, community composition, deterministic process, host preferences, phyllosphere

## Abstract

Deciphering the relationships between microbes and their host plants is critical for a better understanding of microbial diversity maintenance and community stability. Here, we investigated fungal diversity and community assembly in the phyllosphere and rhizosphere of 13 tree species in a subtropical common-garden experiment. The results showed that fungal community structures significantly differed across compartments (leaf, root, and soil) and different tree species. Higher α-diversity was observed in the phyllosphere than in the roots and rhizospheric soil. Fungal community composition (β-diversity) was significantly affected by both compartment and species identity. The fungal community compositions were significantly correlated with soil pH in the roots and the soils as well as with soil nitrate and leaf total phosphorus in the leaves. We found that fungal community assemblies were mainly driven by deterministic processes, regardless of compartments. Moreover, host preference analyses indicated that stronger plant/fungus preferences occurred in leaves than in roots and soils. Our results highlight the differences in tree mycobiome between aboveground and belowground compartments and have important implications for the promotion of biodiversity conservation and management sustainability for the subtropical forest.

**IMPORTANCE** Subtropical mountain forests are widely distributed in Southern China and are characterized by high biodiversity. The interactions between plants and fungi play pivotal roles in biodiversity maintenance and community stability. Nevertheless, knowledge of fungal diversity and of the community assembly patterns of woody plants is scarce. Here, we investigated fungal diversity and community assembly in the phyllosphere and rhizosphere of 13 tree species in a common-garden experiment. We found that both compartment and plant identity influenced fungal diversity, community, and guild compositions, while deterministic processes mainly governed the fungal community assembly, especially in the rhizospheric fungal communities. Our results demonstrate that tree leaves represent stronger host/fungi preferences than do roots and soils. Together, our findings enhance the understanding of the roles of compartment and plant identity in structuring fungal communities as well as promote fungal diversity maintenance in subtropical mountain forest ecosystems.

## INTRODUCTION

The interactions between plants and their inhabiting microbiomes play pivotal roles in biodiversity maintenance, community stability, and ecosystem functioning (1–3). For example, microbial communities are essential to maintaining soil homeostasis and to improving host productivity via the production of secondary metabolites (4, 5), the suppression of pathogens (6, 7), and the promotion of plant growth (8). However, different compartments provide various ecological niches, which are inhabited by highly distinct microbial communities (9), though the microbial diversity, community compositions, and assemblies among different compartment niches are still less documented. In order to improve our ability to predict the responses of ecosystem functions (10), it is crucial to seek a better understanding of the relationships between plants and microbes.

Fungi are one of the most important components of plant microbiota, and they mediate vital ecological functioning, such as soil carbon cycling. Fungi can fundamentally influence and shape ecosystems by producing and transporting nutrients through the food web across multiple trophic levels (11, 12). Interactions among different fungal guilds modify soil carbon dynamics and nutrient availabilities through soil priming effects (13) and the ‘Gadgil’ effect (14). Fungal functions are intrinsically determined by fungal biodiversity and their ecological assembly (10, 12). Therefore, it is essential to decipher fungal biodiversity maintenance and community assembly, which are both key to an improved mechanistic understanding of fungal ecological functioning (15, 16). A number of previous studies have focused on fungi associated with herbaceous plants or crops (17–20), but the fungal diversity and community assembly patterns of woody plants remains less documented.

The aboveground (e.g., leaf) and belowground (e.g., root) compartments have been widely recognized as important determinants of fungal communities in agricultural and grassland ecosystems. For example, Sun et al. (21) found that sorghum’s fungal diversity and community compositions significantly differed across different compartments. Yang et al. (22) reported that the effects of grazing on the fungal community structures of Leymus chinensis were dependent on compartments. Similarly, the effects of different compartment niches on fungi have been demonstrated in other ecosystems, including the plant epiphytic and endophytic phyllosphere in tropical mangrove forests (23) and the root-associated habitats in temperate montane forests (24). Each compartment supplies a unique ecological niche and possesses a highly distinct fungal community. For example, Fusarium, Ilyonectria, Cladosporium and Aspergillus were observed to have a distinctive abundance in the rhizosphere and phyllosphere of Vaccinium macrocarpon (25). Fungal Clitopilus and Mortierella were significantly enriched in the soil and root endosphere samples of the *Cannabis sativa* plant species compared with those of the aerial parts (9), and Phyllosticta vaccinii and P. elongata were frequently isolated from the developing ovaries of cranberry (26). All of these studies indicate that niche differentiation can result in compositional and structural variations in microbial communities across different plant-related compartments. However, most previous studies on fungal community structure focused on one compartment, and fewer efforts have been devoted to understanding the mycobiome across different tree-related compartments in forest ecosystems.

Fungal community structures are generally strongly shaped by plant species identity. For example, Yao et al. (23) demonstrated that plant identity exerted a greater effect on endophytic fungi than on epiphytic fungi in the leaves of mangrove species, revealing the important role of host plant identity in driving phyllosphere epiphytic and endophytic fungal communities. Li et al. (27) highlighted that the root-associated total and saprotrophic fungal richness as well as the total, saprotrophic, and ectomycorrhizal (EM) fungal community compositions were structured by tree identity, but not by plant species diversity. Recently, Zuo et al. (28) found that plant species strongly impacted the niche-based processes of endophytic fungi across the root, stem, and leaf compartments of five xerophyte shrubs in a desert ecosystem, which emphasized the significant host preferences and tissue specificity of endophytic fungi. These findings indicate that different host plants are often characterized by specific fungal community composition and diversity. However, the influences of tree identity on the diversity and community assembly process of fungi in aboveground and belowground compartments have rarely been simultaneously investigated, especially in the subtropical forest ecosystem.

Subtropical mountain forests are widely distributed in south and east China, and they are characterized by a high diversity of plant species that provide habitats for diverse fungi. In this study, based on a common-garden experiment established in a Chinese subtropical forest, we examined fungal communities associated with the leaves, roots, and soils of 13 typical tree species based on Illumina NovaSeq high-throughput sequencing of the ITS2 gene. We aimed to reveal differences in fungal diversity and community composition in aboveground (leaf) and belowground (root and soil) compartments across the 13 kinds of plants, compare the fungal community assembly patterns across different compartments, and identify the host preferences of leaf- and root-associated fungi in the subtropical forest. We hypothesized that both plant identity and compartment would significantly affect fungal diversity and community composition and that the fungal community assemblies of the three compartments (i.e., leaf, root, and soil) were mainly driven by deterministic processes because of the plant host selection.

## RESULTS

### Fungal identification and α-diversity.

After quality control, we obtained 7,556,696 quality-filtered sequences from 7,926,444 raw sequences, of which 369,748 (4.7% of the total reads) singletons were removed. Of these high-quality sequences, 3,918,933 sequences were identified into 6,971 fungal operational taxonomic units (OTUs). We performed normalization using 3,004 sequences (3,004 to 256,438 sequences among leaf, root, and soil samples) and finally obtained 5,560 fungal OTUs (462,616 reads), which were classified into 11 phyla. The total fungal communities were dominated by Ascomycota (71.87% of total reads) and Basidiomycota (23.04%), followed by Mucoromycota (4.08%), Rozellomycota (0.68%), Chytridiomycota (0.22%), Mortierellomycota (0.098%), and others (0.024%) (Fig. S1). The rarefaction curves for the three compartments tended to reach an asymptote, reflecting that the majority of the distinct fungal OTUs had been recovered (Fig. S2).

**FIG 1:**
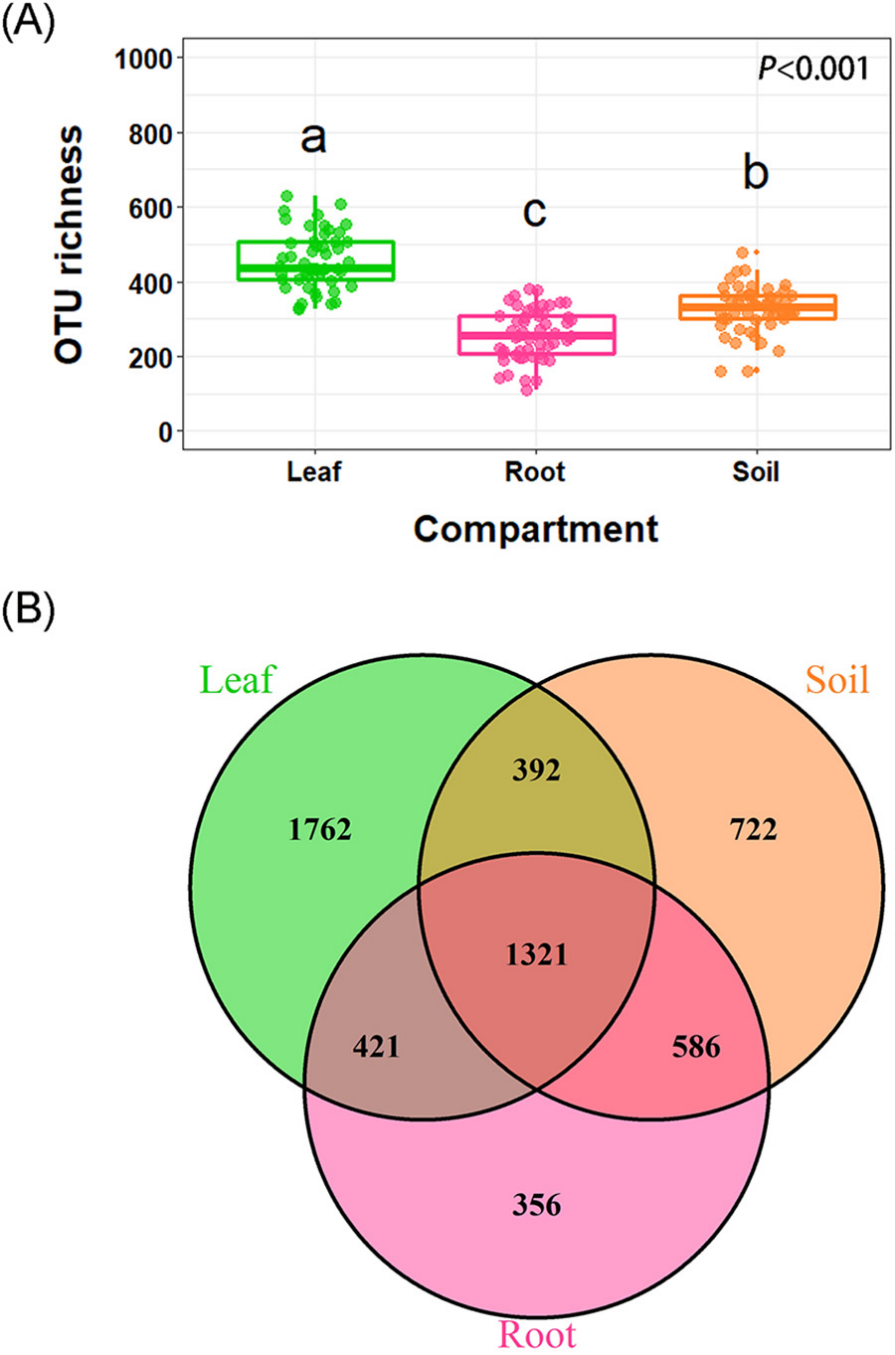
Fungal OTU richness (A) and Venn diagrams (B) comparing the OTU memberships among the three compartments of leaf, root, and soil across 13 tree species in a subtropical forest ecosystem.

**FIG 2:**
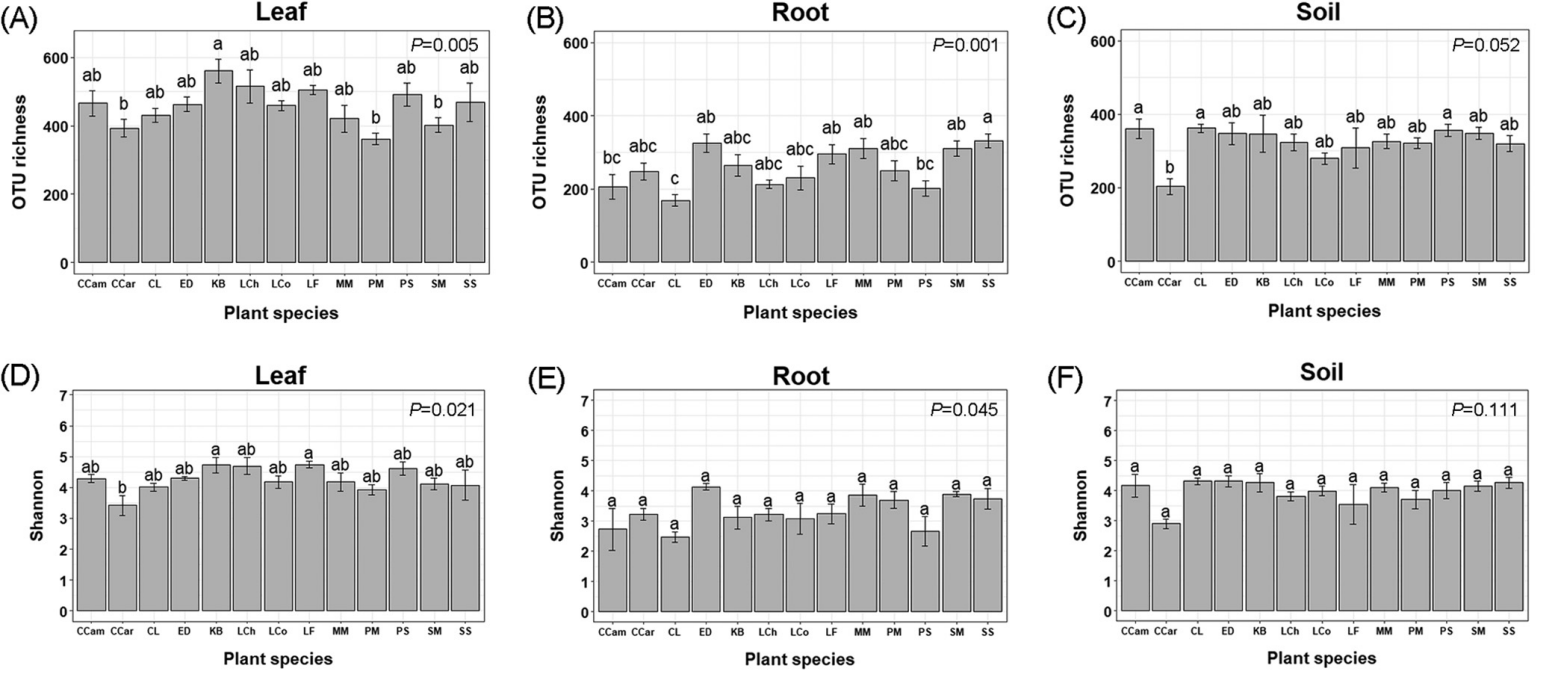
Comparison of OTU richness (A, B, C) and Shannon index (D, E, F) among 13 plant species for each of the three compartments of leaf, root and soil. Different letters above the boxes indicate significant differences at *P* < 0.05. Plant species names: CCam, *Cinnamomum camphora*; CCar, *Castanopsis carlesii*; CL, *Cunninghamia lanceolate*; ED, *Elaeocarpus decipiens*; KB, *Koelreuteria bipinnata*; LCh, *Liriodendron chinense*; LCo, *Lindera communis*; LF, *Liquidambar formosana*; MM, *Michelia macclurei*; PM, *Pinus massoniana*; PS, *Photinia serrulate*; SM, *Sapindus mukorossi*; SS, *Schima superba*.

Two-way analyses of variance (ANOVA) showed that compartment (*F =* 110.4, *P* < 0.001), plant species (*F* = 3.220, *P* < 0.001), and their interaction (*F* = 3.028, *P* < 0.001) had significant effects on the fungal OTU richness, based on the total data set. Plant species had significant effects on the OTU richness of fungi inhabiting leaves (*F* = 3.026, *P* = 0.005), roots (*F* = 4.550, *P* < 0.001), and soils (*F* = 2.003, *P* = 0.052, marginally significant) (Table S1). Additionally, other plant identity-related factors, such as leaf traits (i.e., evergreen broadleaf, deciduous broadleaf, and coniferous leaf), mycorrhizal type (ectomycorrhizal versus arbuscular mycorrhizal), and mean leaf area, also had impacts on fungal α-diversities, including OTU richness as well as the Shannon, Simpson, and Pielou indices (Table S1). The highest and lowest fungal OTU richness were detected in tree leaf and root, respectively (Fig. 1A). A majority of OTUs were detected in two or more compartments. However, higher number of unique fungal OTUs were detected in leaf compartments (1,762) than in soil (722) and root (356) compartments (Fig. 1B). The OTU richness obtained from the plants’ leaves, roots, and soils were different across the 13 plant species (Fig. 2A to C). Significant differences in the Shannon index was detected among the 13 plants for the leaf data set, specifically among a few plants, such as Castanopsis carlesii (CCar) and Koelreuteria bipinnata (KB) (Fig. 2D), but we did not observe any significant differences among the Shannon indices for the soil and root data sets (Fig. 2E and F). Similarly, significant differences in the Simpson and Pielou indices were observed in the leaf (Fig. S3A, D) but not in the root (Fig. S3B, E) or soil (Fig. S3C, F) fungal data sets across the 13 plant species.

### Fungal community and guild composition.

**FIG 3:**
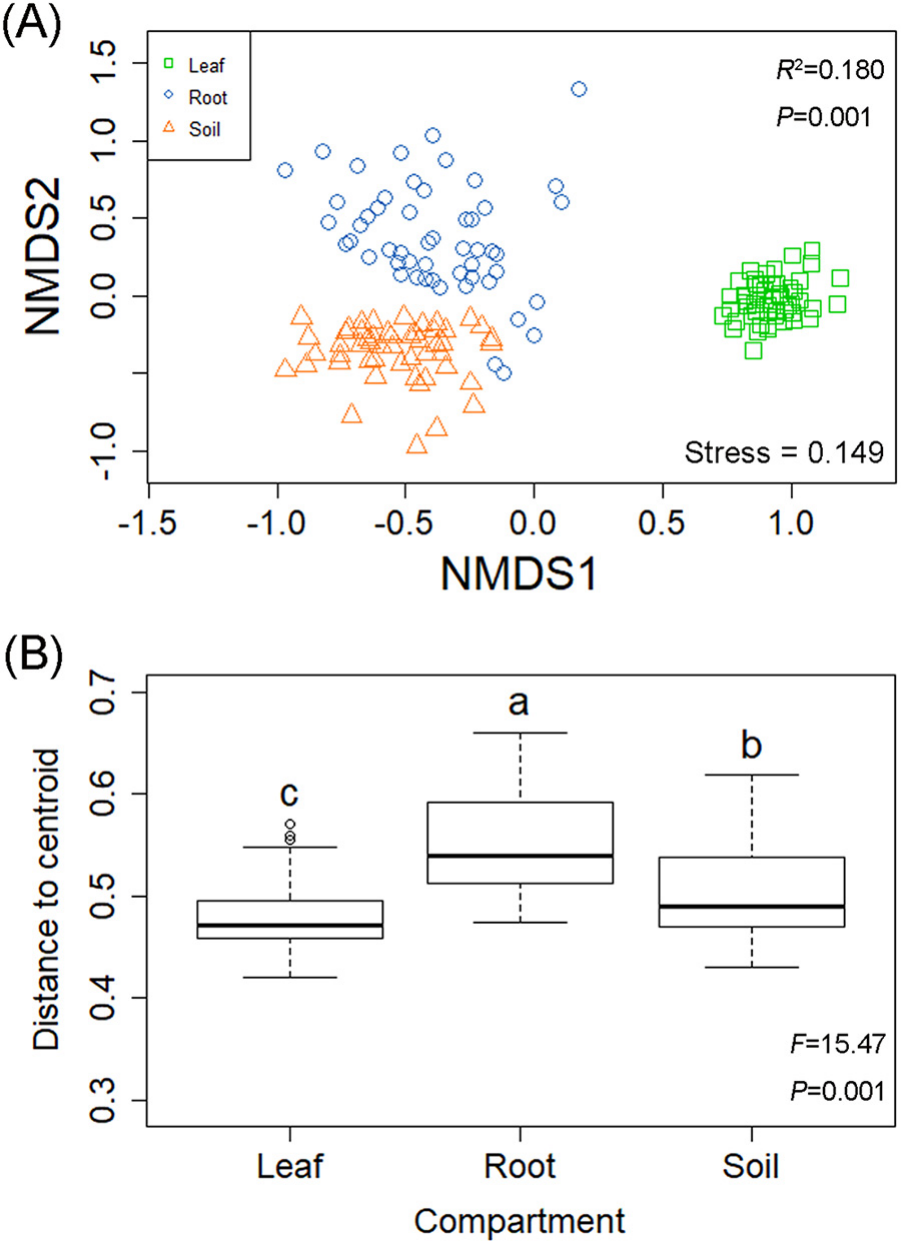
The NMDS ordination showing the fungal community composition among the three compartments of leaf, root, and soil (A) and the results of a beta dispersion analysis to show the fungal assembly extent (B). *R*^2^ and *P* values were obtained from the PERMANOVA (A), and *F* and *P* values were obtained from the ANOVA based on the beta dispersion analysis results (B). Bars without shared letters denote significant differences among the three compartments (B).

**FIG 4:**
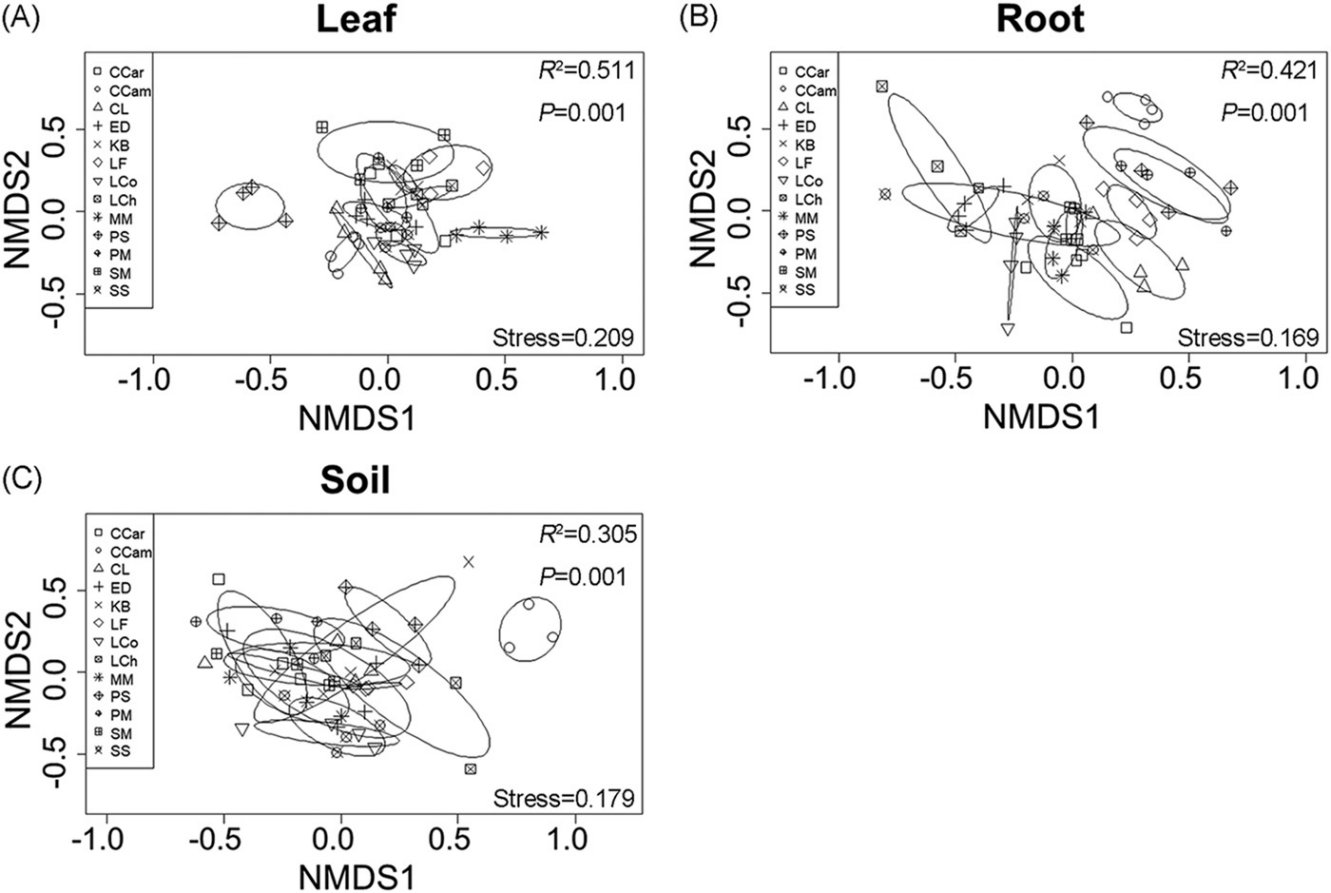
Non-metric multidimensional scaling (NMDS) ordinations of the fungal community compositions in 13 plant species within each of the three compartments of leaf (A), root (B), and soil (C). Ellipses indicate 95% confidence intervals around the centroids of the different plants. *R*^2^ and *P* values represent the results of a PERMANOVA (adonis). Plant species names: CCam, *Cinnamomum camphora*; CCar, *Castanopsis carlesii*; CL, *Cunninghamia lanceolate*; ED, *Elaeocarpus decipiens*; KB, *Koelreuteria bipinnata*; LCh, *Liriodendron chinense*; LCo, *Lindera communis*; LF, *Liquidambar formosana*; MM, *Michelia macclurei*; PM, *Pinus massoniana*; PS, *Photinia serrulate*; SM, *Sapindus mukorossi*; SS, *Schima superba*.

The non-metric multidimensional scaling (NMDS) analysis showed that the fungal community structures in the three compartments clearly differed from each other (Fig. 3A), and this was supported by PERMANOVA results (*R*^2^ = 0.180; *P* < 0.001) (Table S2). Beta dispersion analyses showed that the fungal communities in the roots displayed higher dispersion than did the fungal communities in the soils and the leaves (Fig. 3B, Table S3). Moreover, significant differences in fungal community composition were also observed across the 13 plant species, regardless of compartment (Fig. 4). More specifically, the fungal community compositions in the leaf of Photinia serrulate (PS), in the root of CCar, and in the soil of Cinnamomum camphora (CCam) were significantly distinguishable from other plant species’ corresponding compartments. Our results also showed that compartment niche had a stronger effect (*R*^2^ = 0.180) on fungal community composition than did plant identity (*R*^2^ = 0.138), while their interaction presented the strongest effect (*R*^2^ = 0.518) (Table S2). In addition, fungal community compositions were significantly influenced by leaf traits, mycorrhizal type, and plant leaf area, but these effects were relatively minor compared with the effects of compartment and plant species (Table S2).

In this study, 644 (11.6% of the total), 1,043 (18.7%), and 578 (10.4%) fungal OTUs were identified as fungal (functional) guilds of pathogens, saprotrophs, and symbiotrophs, respectively. The PERMANOVA and NMDS results indicated that the OTU composition of each fungal guild was significantly influenced by compartment (all *P* = 0.001) (Fig. S4). The symbiotrophic fungal community (*R*^2^ = 0.240, adonis) was more significantly different among compartments than were the pathogen (*R*^2^ = 0.215) and saprotrophic (*R*^2^ = 0.166) fungal communities. Moreover, symbiotrophic fungal community composition was more convergent (meaning that the points assembled more closely) than the other two guilds (Fig. S4). The NMDS results indicated that the compositions of pathogens, saprotrophs, and symbiotrophs exhibited differential separation across the 13 tree species, irrespective of compartments (Fig. S5). Moreover, it was suggested that plant identity exerted a significant effect on the community composition of each fungal guild, according to the PERMANOVA results (all *P* < 0.05) (Fig. S5).

### Preferences of plants and fungi.

**FIG 5:**
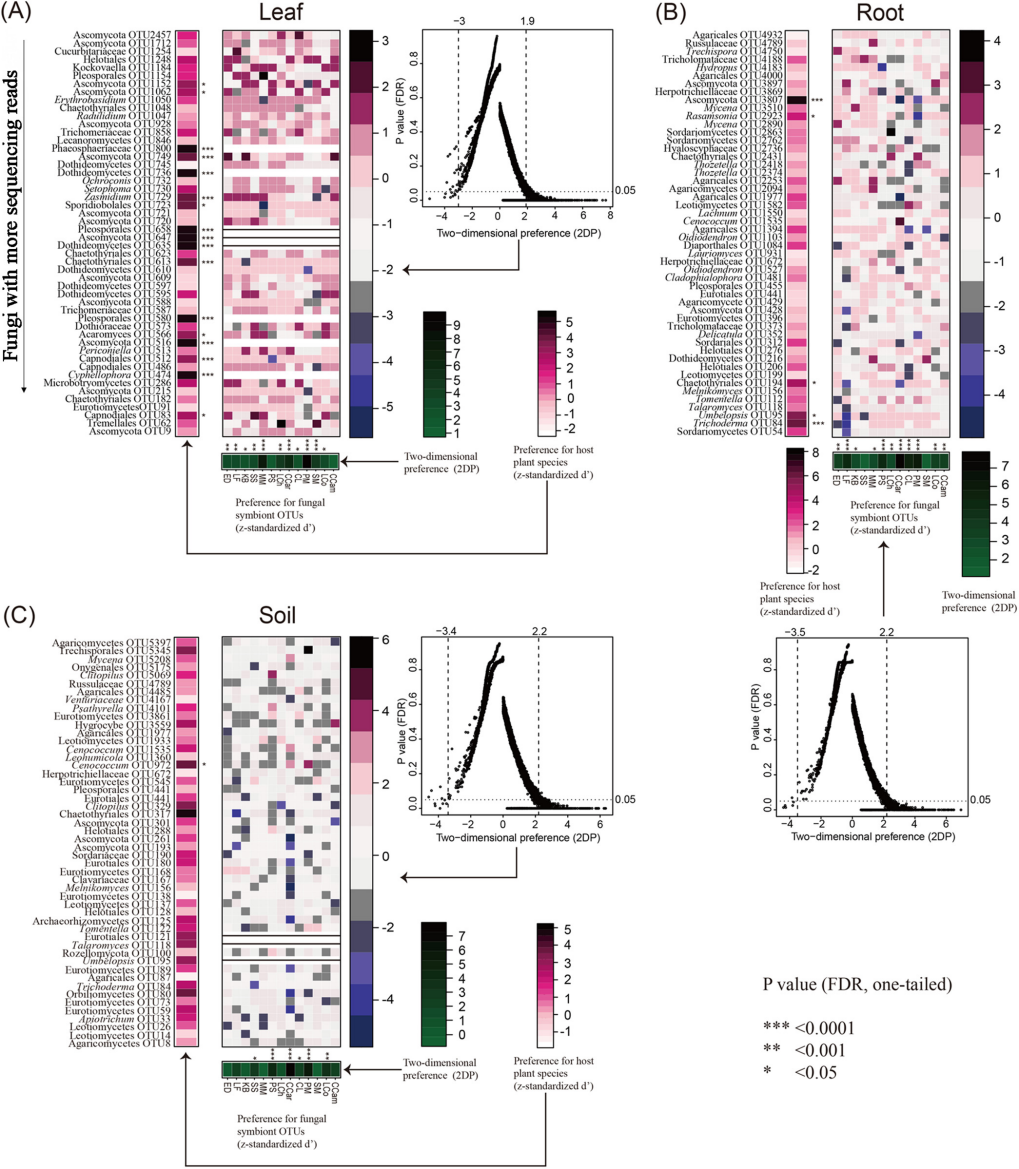
Preferences observed in plant-leaf (A), plant-root (B), and plant-soil (C) fungal associations. The standardized *d’* estimate of preferences for fungal OTUs is shown for each plant species (column). Likewise, the standardized *d’* estimate of preferences for plant species is indicated for each of the observed fungal OTUs (row). Each cell in the matrix indicates a two-dimensional preference (2DP) estimate, which measures the extent to which the association of a focal plant-fungal pair was observed more or less frequently than would be expected by chance. Black lines indicate that the two-dimensional preference (2DP) between a fungus and a plant was not available (NA). The *P* values were adjusted based on the false discovery rate (FDR).

Plant-fungi preference analyses showed that, respectively, 11, 11, and 6 out of the 13 tree species preferred specific leaf, root, and soil fungal OTUs (Fig. 5). In the leaf fungal communities, 34% (17 OTUs) of the abundant OTUs displayed significant host plant preferences, such as OTU580 (Pleosporales), OTU647 (Ascomycota), OTU736 (Dothideomycetes), and OTU800 (Phaeosphaeriaceae) (Fig. 5A). In the root fungal communities, 10% (5) of the abundant OTUs exhibited significant host preferences, such as OTU84 (*Trichoderma*) and OTU3807 (Ascomycota) (Fig. 5B). One abundant OTU (OTU972, *Cenococcum*) from the soil compartment had significant host preferences (Fig. 5C).

We found that, respectively, 4%, 20%, and 16% of the pairs of tree species and abundant fungal OTUs for the leaf, root, and soil compartments exhibited remarkably strong preferences. For example, the pairs of plant M. macclurei and leaf fungal OTU512 (Capnodiales), C. carlesii and leaf OTU613 (Chaetothyriales), P. serrulate and root OTU84 (*Trichoderma*), C. lanceolate and root OTU3807 (Ascomycota), and P. serrulate and *Pinus massoniana* with soil OTU972 (*Cenococcum*) had strong plant-fungus preference relationships (Fig. 5). The results further suggested that tree identity exerted impacts on the fungal community composition in tree leaves, roots, and rhizospheric soils. The host trees selected specific fungi, and in return, the fungi preferred to select their specific host partners.

### Fungal biomarkers.

A LEfSe analysis showed that one order, three families, three genera, and six species of fungi identified from the leaf compartment, one family, three genera, and two species from the root compartment, and one order, four families, three genera, and four species from the soil compartment exhibited significantly different relative abundances (Fig. S6A). For example, the fungal order Thelebolales, family Pseudeurotiaceae, genus *Hyphozyma* and species Hyphozyma roseonigra were enriched in leaves, while the fungal family Auriculariaceae and the genera *Calonectria*, *Gymnopilus*, and *Rasamsonia* were enriched in roots, and the fungal order Onygenales, family Trichosporonaceae, genus *Mortierella,* and species Oidiodendron chlamydosporicum were enriched in soils (Fig. S6B).

### Community assembly and drivers.

**FIG 6:**
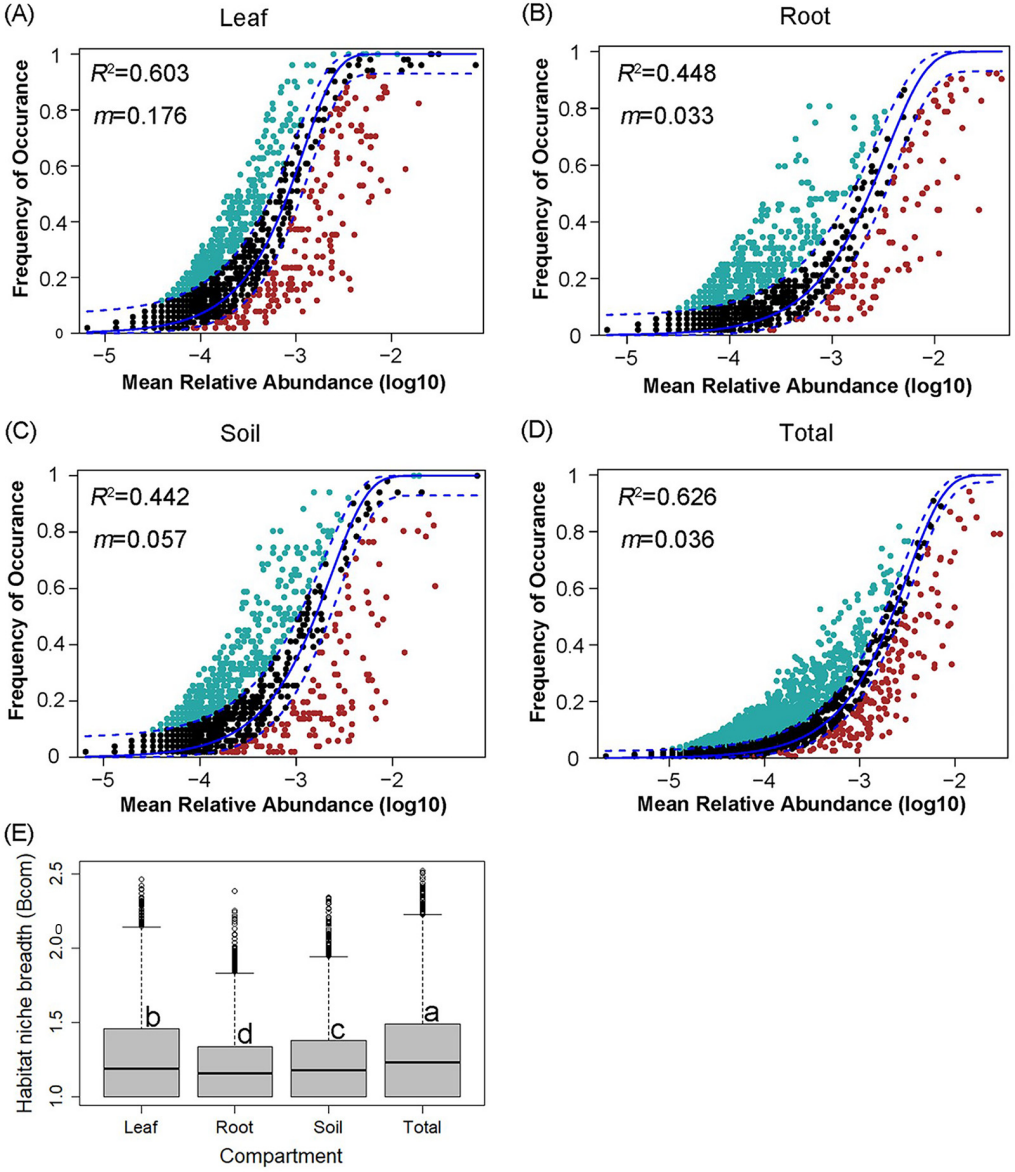
The neutral community model shows that the neutral interpretation has a good fit to fungal community distribution for the leaf (A), root (B), soil (C), and total (D) fungal data sets. The predicated occurrence frequency is shown as a solid blue line, and dashed blue lines represent 95% confidence intervals around the model prediction. Red and green dots indicate the fungal operational taxonomic units that occur less and more frequently than given by the model. *R*^2^ and *m* indicate the degree of fitting to the neutral community model and the immigration rate, respectively. The comparison of the mean habitat niche breadth (*Bcom*) in all taxa among the three compartments (subsets) and the total fungal community (Kruskal-Wallis test) is shown together (E).

The neutral community model showed that the goodness-of-fit was different among the compartments of tree leaf (*R*^2^ value indicating 60.3% variability explained), root (44.8%), and soil (44.2%) as well as the total data set (62.6%) (Fig. 6). The migration rate (*m*) was consistently low (0.033 to 0.176, compared to 1), reflecting strong deterministic processes driving the fungal community assemblies of the four data sets (Fig. 6). Moreover, the migration rates were lower in the root (*m* = 0.033) and soil (*m* = 0.057) compartments than in the leaf (*m* = 0.176) compartment. We also calculated the community-level habitat niche breadths (*Bcom*) to explore the relative importance of deterministic and stochastic processes in fungal community assembly. Our results indicated that a significantly higher *Bcom* value was observed for leaf fungi than for soil and root fungi (Fig. 6E). Mantel tests showed that fungal community compositions were significantly correlated with soil and leaf properties (Table 1). The leaf fungal community was significantly correlated with the soil NO_3_^–^-N (*r* = 0.137, *P* = 0.049) and leaf TP (*r *= 0.193, *P* = 0.009), while the root (*r* = 0.173, *P* = 0.013) and soil (*r* = 0.214, *P* = 0.003) fungal communities were significantly affected by the soil pH.

**TABLE 1:** Results of partial Mantel tests (*r* and *P* values) between the fungal community matrix of each compartment and matrices of soil (leaf) parameters

Factors	Leaf		Root		Soil	
Soil (leaf)	*r* value	*P* value	*r* value	*P* value	*r* value	*P* value
pH	0.086	0.124	0.173	**0.013**	0.214	**0.003**
TOC	−0.042	0.657	−0.021	0.542	−0.004	0.477
NH_4_^+^-N	−0.093	0.895	0.006	0.486	0.111	0.094
NO_3_^–^-N	0.137	**0.049**	0.016	0.423	−0.024	0.549
AP	−0.032	0.67	0.102	0.095	−0.051	0.731
TC	−0.098	0.972	0.073	0.105	0.028	0.302
C/N	−0.030	0.639	−0.138	0.995	−0.03	0.637
TN (Leaf)	(−0.105)^*a*^	(0.913)	−0.074	0.883	0.004	0.437
TP (Leaf)	(0.193)	(**0.009**)	0.032	0.274	−0.013	0.576
N/P (Leaf)	(0.028)	(0.343)	−0.013	0.478	0.036	0.301

a The values within parentheses were calculated using the plant leaf parameter, including nitrogen (N) and phosphorus (P) concentrations as well as the ratios of N to P. The *P* values indicative of statistical significance are shown in bold.

## DISCUSSION

### Fungal α-diversity was different across compartments.

Based on the investigation of fungi living in the phyllosphere and rhizosphere of 13 tree species in a subtropical plantation forest, we demonstrated that fungal diversity was significantly different among the leaf, root, and soil compartments. The fungal α-diversity in the tree phyllosphere was higher than that observed in the root-associated (root and soil) compartments (Fig. 1). In contrast, previous studies demonstrated that the soil compartment harbored a higher fungal α-diversity than did the other compartments (9, 21). However, compared with the soil compartment, higher α-diversities were observed in the root endosphere and phyllosphere compartments (23, 29). In this study, the lower fungal diversity in the soil compared to that of the leaf phyllosphere may be attributable to the fact that the soil samples were collected from rhizosphere, in which fungal components were environmentally filtered by the host selection effect (3). Moreover, the higher aboveground heterogeneity (size, height, and spatial distance) of the plant leaves for the 13 tree species might result in a greater fungal diversity than that of the rhizosphere compartments, which have relatively high habitat homogeneity. In addition, the relatively high fungal diversity detected in the leaf compartment may be attributable to the larger amounts (5.0 g) of leaf samples used for analyzing the phyllospheric fungi.

### Plant identity influenced fungal α-diversity.

In our study, plant species had a strong effect on the total fungal α-diversity (Table S1). Additionally, we found that tree species had strong effect on the fungal OTU richness of the leaf and root compartments (Fig. 2), a result supported by previous findings that plant identity significantly influences fungal richness in the phyllosphere (30) and the roots (27). Plant species exerted a marginally significant (*P* = 0.052) effect on soil fungal OTU richness. These results indicated that plant identity had a strong impact on the fungi filtered by the host selection effect. Mycorrhizal type is another important identity factor that may have a significant influence on fungal α-diversity, supported by the results of Singavarapu et al. (31). This may be because different functional groups of fungi were recruited by different mycorrhizal plants (32). Previous studies illustrated that the fungal diversities were significantly different between deciduous and evergreen forests (33), with higher α-diversity being detected in the leaves of evergreen trees (34, 35). In this study, our results also showed that leaf traits and mean leaf area could affect the fungal α-diversity in the phyllosphere, rather than in the root and soil compartments.

### Community composition was determined by compartment and plant identity.

The fungal community compositions were significantly different across the three compartments of leaf, root, and soil (Fig. 3). Similar results with sorghum and hemp were reported in previous studies (9, 21). Different compartments have disparate physicochemical properties due to structural differences and exposure to different environmental conditions (36), which may result in the selective recruitment of microbial communities (21, 37). Our results showed that plant identity (tree species, leaf trait, mycorrhizal type, and mean leaf area) had strong effects on the total, leaf, root, and soil fungal structures (Table S2), which are in line with the results reported by Li et al. (27) and Kambach et al. (38). The community compositions of symbiotic, saprotrophic, and pathogenic fungi were consistently influenced by tree species identity, and this result is also supported by previous studies (27, 39). Plant identity effects on fungal community compositions may be attributable to host/fungus preferences and/or host selections. Host/fungus preferences mean that the host plant selects specific fungi, and the fungi, in return, select a specific host plant. Host selection indicates that plants can recruit or filter microbes in the rhizosphere and phyllosphere from the soil, which serves as a primary reservoir for the plant-associated microbiome (3, 9). Our results provided evidence that host preferences or host selections could influence tree-related fungal community composition in forest ecosystems.

We found that fungal community assembly was mainly driven by a deterministic process, rather than a stochastic process, across the three compartments of leaf, root, and soil. Similarly, previous studies demonstrated that soil fungal community assemblies were strongly shaped by deterministic (niche-based) factors (e.g., [40, 41]). For root-associated fungal communities, deterministic processes dominated at most of the sites (42), while neutral processes still had a minor influence on community assembly and might be important in spatially isolated communities as well as those with strong gradients of fungal diversity (42, 43). According to the lower migration rate of the leaf compartment than that of the root and soil compartments, our results suggested that fungal communities in the phyllosphere were less affected by deterministic process than were those in the other two compartments.

We consider that there are two reasons for this explanation. First, common anthropogenic modifications can interactively control fungal community assembly (44), In this case, the phyllosphere habitat is more susceptible to anthropogenic influences compared to the root and soil habitats. Second, when the environmental conditions are spatially heterogeneous, increased stochasticity can reduce the importance of deterministic filters (44), and local environmental filtering (i.e., climate and/or plant traits) could have an important role in structuring fungal communities (45). Therefore, stochastic factors, such as random airborne dispersal, random disturbances, the priority effect, and atmospheric environmental conditions, such as solar radiation, humidity, and temperature, are critical for shaping the fungal communities that inhabit plant leaves (46).

### Biomarkers were different among compartments.

More fungal biomarkers were detected in the phyllosphere compartment than in the roots or rhizospheric soil, according to the LEfSe analysis, which was in line with a previous study (3). These results provided comprehensive empirical evidence for host selection and a theoretical framework of coevolution between the host plants and the microbes, in which the plants employed exudations to recruit, filter, and enrich fungal taxa with specific functions in different niches (3, 47). The phyllosphere compartment represents the majority of biomarker species, possibly because the phyllosphere fungi are more functionally differentiated than those the in root and soil compartments. The fewest biomarkers were detected in the root compartment, which could be explained by the fact that there were intensive selection pressures caused by the host immune system and plant exudates in the root endosphere, and thus relatively few fungi were able to colonize therein (3, 48). Particularly, fungi Ochroconis cordanae and *Rasamsonia* were the potential biomarkers with the highest enrichment in the leaf and the root compartments, respectively. In fact, O. cordanae was introduced by Giraldo et al. (49), having been isolated from plant leaves, and most *Ochroconis* species isolated from leaves were plant pathogens (50). The genus *Rasamsonia*, belonging to fungal family Trichocomaceae, order Eurotiales, was similarly identified as a rhizospheric biomarker in two recent studies (51, 52). These results indicated that the fungal biomarker species were distinct across different compartments and that their formation and occurrence might be related to host plant selections and environmental filtering processes (3, 53). However, more direct evidence is needed to reveal the relationships between fungal biomarkers and host or environmental conditions.

### Host/fungi preference differed across compartments.

Differences in host preference and/or specificity among fungi probably resulted from some complicated factors, including the intimacy of association, phylogenetic and physiological differences among hosts, competitive interactions among fungi, mutualistic effects, and preferential allocation of resources between symbionts (54, 55). Indeed, our results demonstrated that there were, respectively, 11, 11 and 6 of the 13 tree species showing significant preferences for fungi in the leaf, root, and soil compartments. Plants represent stronger preferences for leaf- and root-associated fungi than for fungi in soil habitats, suggesting the strong selection pressures caused by the host immune system to allow more fungi in different niches to penetrate and colonize (3). We found that all four of the deciduous broad-leaved tree species (i.e., Koelreuteria bipinnata, Liriodendron chinense, Liquidambar formosana, and Sapindus mukorossi) showed significant preferences for fungi only in the leaf compartment. Previous studies suggested that broad‐leaved forest types could affect soil fungal community structure (56). Two coniferous species of C. lanceolate and P. massoniana consistently showed significant preferences for fungi, irrespective of compartments, suggesting the important effects of tree traits on host preferences for fungal partners.

On the other hand, the preferences of fungi for plants were significantly distinct across different compartments. In this study, the more abundant leaf fungi (number = 17) showed significant plant preferences over those in the root (number = 5) and soil (number = 1) compartments (Fig. 5). This might be because the differences in host chemistry (e.g., nutrient contents) could fundamentally influence fungal colonization and could consequently result in distinctly dominant fungal components across compartments (23, 57). Specifically, the leaf-associated fungal members of *Cyphellophora* (OTU474 detected in this study) and *Zasmidium* (OTU729) showed significant host preferences in the leaf compartment, which had been always isolated from plant leaves and been considered causal agents for citrus greasy spot (58). The fungal *Trichoderma* (OTU84, from the roots in this study) species was compatible with the relatively stable microenvironments for plant roots and might provide a range of benefits to their hosts (59, 60). The EM fungus *Cenococcum* (OTU972, from the soil habitat in this study) can form black, round to irregular sclerotia and can commonly exist in forest soils (61). Our results clearly showed that most of these OTUs (87%) were belonging to Ascomycota, which would explain why Ascomycota was widely distributed in various habitats and showed higher species diversity and a faster evolutionary rate (62).

### Conclusions.

In summary, we investigated fungal diversity, community composition, and assembly patterns in both aboveground (leaf) and belowground (root and soil) compartments across 13 tree species in a subtropical forest ecosystem. Both compartment and plant identity significantly affected fungal diversity, community, and guild compositions, but compartment niches exerted stronger impacts on fungal community structures. Our results also indicated that deterministic process mainly governed the fungal community assembly and that phyllosphere fungal community assemblies were less impacted by deterministic processes than those in root and soil compartments. Furthermore, it was showed that tree species preferred specific leaf, root, and soil fungal OTUs and that the leaf compartment represents stronger host/fungi preferences than do the root and soil compartments. Taken together, although we acknowledge that the short fungal barcodes (<500 bp) may provide limited information for species discrimination and/or taxonomic assignment, our findings advance the understanding of the roles of compartment and tree identity in structuring fungal communities, which is critical to promoting microbial diversity maintenance and management sustainability for the subtropical forest ecosystem.

## MATERIALS AND METHODS

### Sites, experimental design, and sampling.

This study was conducted at Sanming Forest Ecosystem National Observation and Research Station (26°19′55′′N, 117°36′53′′E), Fujian Province, China. This region has a mean annual temperature and precipitation of 19.5°C and 1,700 mm, respectively. The soil is classified as typical granite yellow or red soil. A common-garden was established after clear-cut logging with control-burning before tree planting. The experiment was set up using a randomized block experiment design. Briefly, the whole garden was divided into four regions (replicates). In each region, 13 plots (10 × 10 m^2^) were randomly distributed for the planting of each of 13 tree species: Cinnamomum camphora (CCam); Castanopsis carlesii (CCar); Cunninghamia lanceolate (CL); Elaeocarpus decipiens (ED); Koelreuteria bipinnata (KB); Liriodendron chinense (LCh); Lindera communis (LCo); Liquidambar formosana (LF); Michelia macclurei (MM); Pinus massoniana (PM); Photinia serrulate (PS); Sapindus mukorossi (SM); Schima superba (SS). A total of 100 trees of each species were evenly planted in each plot in February, 2012.

Soil, plant root, and leaf samples were collected in October 2020. From each plot, three sample types (leaf, root, and soil) were collected from three randomly selected individuals (*c.* 10 m between each other) of the same tree species, which resulted in a total of 156 composite samples from 52 plots. Within each plot, 10 healthy leaves were randomly collected, and one root sample (~100 g) was collected by tracing roots from the base of the tree trunk. The rhizospheric soil sample (~100 g) from each plant individual was simultaneously collected with the root sampling. All of the samples were transported in an ice box to the laboratory. Soil samples were sieved through a 2 mm sieve to remove roots and debris, then stored at 4°C prior to the analyses of the soil physicochemical properties, then frozen at −80°C for molecular analyses. Leaf and root samples were stored at −80°C prior to DNA extraction.

### Plant and soil properties.

Soil samples were analyzed for the following soil properties: soil pH, total carbon (TC), total nitrogen (TN), total organic carbon (TOC), mineral nitrogen (NO_3_^−^-N and NH_4_^+^-N), total phosphorus (TP), and available phosphorus (AP). Leaf total nitrogen and leaf total phosphorus were tested as leaf properties. Briefly, soil pH was determined with a soil-to-water ratio of 2:5 (wt/vol) using a glass electrode (FE20, Mettler Toledo). Soil total carbon (TC) and total N (TN) were determined with an Elementar Vario EL III (Elementar Analysensysteme GmbH, Germany). Soil total organic carbon (TOC) was determined using the K_2_Cr_2_O_7_ oxidation-reduction titration method. Soil mineral N (NH_4_^+^-N and NO_3_^−^-N, extracted with 2 M KCl) and soil total P (TP, extracted with H_2_SO_4_:HClO_4_ [4:1, vol/vol] solution) were determined using a continuous flow analyzer (SAN++, Skalar, Netherlands) (63). Soil available P (AP) was determined using the Mehlich-3 method (64). Leaf N and P concentrations were determined using the standard micro-Kjeldahl and vanadomolybdo phosphoric acid yellow color methods, respectively (65).

### DNA extraction, PCR and sequencing.

Phyllospheric fungi were washed from the leaf surfaces according to Gourion et al. (8). Tree leaves (5.0 g) were transferred into a 50 mL plastic tube filled with sterile, cooled TE buffer and subjected to alternating sonication (45 s) and vortexing (30 s), three times. The washing solution was passed through sterilized medical gauze and a 0.22-μm filter via a Vacuum Filtration System (MultiVac610-MS-T, Rocker, China). Root samples were surface cleaned with water to remove soil, washed with sterile water three times, and then homogenized using a sterilized mortar and pestle after freeze-dried treatment using liquid N_2_. We extracted total DNA from all leaf phyllosphere (i.e., 0.22 μm filters), root, and soil samples using the PowerSoil DNA isolation kit (MoBio Laboratories, Inc. USA), according to the manufacturer’s instructions. The DNA concentration was measured using a NanoDrop 1000 Spectrophotometer (Thermo Scientific, Wilmington, USA).

The PCR primers of 5.8S-Fun (5′- AACTTTYRRCAAYGGATCWCT -3′) and ITS4-Fun (5′- AGCCTCCGCTTATTGATATGCTTAART -3′), linked with a 12-base barcode, were used in this study. Thermocycling conditions were as follows: 95°C for 3 min, followed by 35 cycles of 30 s at 94°C, 40 s at 58°C, and 1 min at 72°C. Each DNA sample was amplified in triplicate. The PCR products were purified using the Wizard SV Gel and PCR Clean-Up System (Promega, San Luis Obispo, USA) and were pooled with equimolar amounts (200 ng) from each sample. After measuring the DNA concentrations via a TBS 380 fluorescence spectrophotometer (Promega, Madison, USA), the purified PCR products were mixed at equimolar amounts and sequenced using an Illumina NovaSeq sequencer (Illumina, San Diego, USA; 2 × 250 bp paired ends) at the Environmental Genome facilities of the Chengdu Institute of Biology, Chinese Academy of Sciences (Chengdu, China).

### Bioinformatics analysis.

Raw sequences were processed using the QIIME 2 pipeline (66) to remove low-quality reads that lacked a valid primer/barcode sequence, contained ambiguous bases, or had an average quality score of <20. Chimera sequences were checked using USEARCH v8.0 software (67). High quality ITS2 sequences were subjected to de-replication and de-singleton, then clustered into operational taxonomic units (OTUs) at a 97% similarity level based on the UPARSE pipeline, using USEARCH. The representative sequence of each OTU was selected through the ‘get al.oturep’ command and was identified using the SINTAX algorithm against the UNITE database (68) with a confidence threshold of 65% (69). To eliminate the potential effects of uneven sequence depths across samples for the fungal community analysis, the number of sequences per sample was rarefied to the smallest sample size using the ‘sub.sample’ command in Mothur 1.32.2 (70). The resultant fungal community matrices were used for the analyses of the fungal α- and β-diversity and community assembly pattern. Fungal OTUs were assigned to functional guilds at the ‘highly probable’ and ‘probable’ levels, following Tedersoo et al. (11) and the most updated list in FUNGuild (71). The raw sequences (files in FASTQ format) were deposited in the National Center for Biotechnology Information under accession no. PRJNA851923.

### Statistical analysis.

Fungal α-diversity was defined as the observed OTU richness, Shannon-Wiener, Simpson’s, and Pielou’s evenness of each sample. A two-way analysis of variance (ANOVA) was used to detect the main effects of compartment and plant identity, as well as their interaction effect, on α-diversities, while the nonparametric Kruskal-Wallis test was used to analyze the data that did not satisfy homogeneity of variance. The significant differences in the microbial α-diversity between different plants were determined using Welch’s *t*-test. The overall comparison of diversity and community composition based on OTU richness was visualized by a Venn diagram using the ‘venn.diagram’ function in the ‘VennDiagram’ package (72). Multifactorial permutational analysis of variance (PERMANOVA) was conducted based on distance matrices using the ‘adonis’ function in the ‘vegan’ package with 999 permutations (73) to evaluate the effects of compartments (total data) and tree species (data subset of each compartment) on fungal β-diversity.

The fungal community composition was ordinated using nonmetric multidimensional scaling (NMDS) with dissimilarity matrices using the ‘metaMDS’ function in the ‘vegan’ package (74). To test the homogeneity of the fungal community among different compartments, the beta dispersion of the Simpson dissimilarity was explored via the ‘betadisper’ function in the ‘vegan’ package (75). Significant differences in the relative abundances of fungal taxa between compartments were identified using the linear discriminant analysis (LDA) effect size (LEfSe), with a logarithmic LDA score of 3.0 as the threshold. This analysis was performed on a Galaxyweb-based interface (http://huttenhower.sph.harvard.edu/galaxy/). Mantel tests were performed to analyze the effects of soil properties on fungal diversity via the “vegan” package.

A neutral community model, proposed by Sloan et al. (76), was used to determine the contribution of stochastic processes to the fungal community assembly by predicting the relationship between the occurrence frequencies of OTUs (the proportion of local communities in which each OTU is detected) and their abundance (the mean relative abundance across all local communities) (41, 76). This model emphasizes the effect of stochastic dispersal and drift but ignores the ecological differences between species and their responses to the surrounding environment. In this model, the estimated migration rate (*m*) was a parameter for evaluating the probability that a random loss of an individual in a local community would be replaced by a dispersal from the metacommunity. When the migration rate (*m*) is close to 0, community assembly tends to be more deterministic, and when *m* is close to 1, it tends to be determined by stochastic process (41, 76). The parameter *R*^2^ represented the overall fit to the neutral model. An *R*^2^ value closer to 1 implied that the community was consistent with the neutral process of dispersal and ecological drift, whereas an *R*^2^ value closer to0 implied a lack of model fit. The two parameters were determined using nonlinear least squares fitting in the ‘minpack.lm’ package (77). In addition, the calculation of 95% confidence intervals around the model prediction was conducted via 1,000 bootstrap replicates in the ‘Hmisc’ package (78). Niche breadth was calculated according to Levin’s niche breadth index (79), and this analysis was conducted using the ‘niche.width’ function in the ‘spaa’ package (80). The host/fungal preferences were evaluated according to the method of Toju et al. (81), using the “bipartite” and “parallel” packages. OTUs with more reads (the top 50) were defined as abundant OTUs, and these were chosen for the plant-fungus preference analyses (79). All statistical analyses were conducted using R-4.1.2 (82). We calculated rarefaction curves using the ‘specaccum’ function in the “vegan” package.

### Data availability.

The raw sequences were deposited in the National Center for Biotechnology Information under accession no. PRJNA851923.
