# Supplementary material for: Compartment and Plant Identity Shape Tree Mycobiome in a Subtropical Forest
Source: Microbiol Spectr. 2022 Jul 12;10(4):e01347-22. doi: 10.1128/spectrum.01347-22 (PMC9430249; doi:10.1128/spectrum.01347-22)
Supplement: Supplemental file 1 — Supplemental material. Download spectrum.01347-22-s0001.pdf, PDF file, 1.4 MB [file spectrum.01347-22-s0001.pdf]

Title page:

Compartment and Plant Identity Shape Tree Mycobiome in a Subtropical Forest

Hao Yang[1], Zhijie Yang[1,2], Quan-Cheng Wang[1], Yong-Long Wang[3], Hang-Wei Hu[1], Ji-Zheng He[1,2], Yong Zheng[1]*, Yusheng Yang[1,2]

[1] Key Laboratory for Humid Subtropical Eco-geographical Processes of the Ministry of Education, Fujian Normal University, Fuzhou 350007, China.

[2] Sanming Forest Ecosystem National Observation and Research Station, Sanming, Fujian 365002, China.

[3] Faculty of Biological Science and Technology, Baotou Teacher's College, Baotou, Inner Mongolia 014030, China.

*Author for correspondence: Dr. Yong Zheng

Tel.: +86 (0)591 8346 5214; *Fax*: +86 (0)591 8346 5397

E-mail: zhengy@fjnu.edu.cn

Running title: Compartment and plant identity shape tree mycobiome

Supplementary Information

Figure legends

FIG S1 The distribution of fungal phyla among total dataset and sub-datasets of three compartments of leaf, root and soil.

FIG S2 Rarefaction curves for observed fungal OTUs recovered from three compartments (A) of leaf (B) and root (C) and soil (D) samples among 13 woody plant species in subtropical forest. Plant species names: CCam, *Cinnamomum camphora*, CCar, *Castanopsis carlesii*, CL, *Cunninghamia lanceolate*, ED, *Elaeocarpus decipiens*, KB, *Koelreuteria bipinnata*, LCh, *Liriodendron chinense*, LCo, *Lindera communis*, LF, *Liquidambar formosana*, MM, *Michelia macclurei*, PM, *Pinus massoniana*, PS, *Photinia serrulate*, SM, *Sapindus mukorossi*, SS, *Schima superba*.

FIG S3 Simpsons index comparison amongst 13 plant species for each of three compartments of leaf (A), root (B) and soil (C), and Pielou index comparison amongst 13 plant species for each of three compartments of leaf (D), root (E) and soil (F). Plant species names: CCam, *Cinnamomum camphora*, CCar, *Castanopsis carlesii*, CL, *Cunninghamia lanceolate*, ED, *Elaeocarpus decipiens*, KB, *Koelreuteria bipinnata*, LCh, *Liriodendron chinense*, LCo, *Lindera communis*, LF, *Liquidambar formosana*, MM, *Michelia macclurei*, PM, *Pinus massoniana*, PS, *Photinia serrulate*, SM, *Sapindus mukorossi*, SS, *Schima superba*.

FIG S4 Non-metric multidimensional scaling (NMDS) of the fungal community compositions for pathogen (A) and saprotroph (B), and symbiotroph (C) fungi. R^2 and P values represent the result of PerMANOVA analysis for each fungal functional guild.

FIG S5 Non-metric multidimensional scaling (NMDS) of the fungal community compositions in 13 plant species within each of three compartments of leaf and root, and soil in pathogen and saprotroph, and symbiosis fungi. (A), (B) and (C) represent pathogenic fungal community compositions of leaf, root and soil, respectively. Similarly, (D), (E) and (F) represent saprotroph fungal community compositions of leaf, root and soil, respectively, and (G), (H) and (I) represent symbiotroph fungal community compositions of leaf, root and soil, respectively. Ellipses indicate 95% confidence intervals around centroids of different plants. R^2 and P values represent the results of PerMANOVA analyses. Plant species names: CCam, *Cinnamomum camphora*, CCar, *Castanopsis carlesii*, CL, *Cunninghamia lanceolate*, ED,

Elaeocarpus decipiens, KB, *Koelreuteria bipinnata*, LCh, *Liriodendron chinense*, LCo, *Lindera communis*, LF, *Liquidambar formosana*, MM, *Michelia macclurei*, PM, *Pinus massoniana*, PS, *Photinia serrulate*, SM, *Sapindus mukorossi*, SS, *Schima superba*.

FIG S6 (A) Linear discriminant analysis (LDA) effect size (LEfSe) showing significant differences in the relative abundances of fungal taxa across leaf (red, 13), root (green, six) and soil (blue, 12), with a logarithmic LDA score of 3.0 as the threshold. (B) LEfSe cladogram indicating the phylogenetic distribution of 19 fungal lineages (above species level). The size of each small circle is roughly proportional to the relative abundance of that given taxa. Six rings of the cladogram represent phylogenetic levels from domain to genus from the inside outwards. The small yellow circles represent the fungal taxa with no significant differences, while small circles and sectors with other different colors represent the significantly enriched fungal taxa in different compartments. Biological classifications are listed beside the cladogram, 'o' refers to order, 'f' refers to family, 'g' refers to genus and 's' refers to species.

Fig. S1

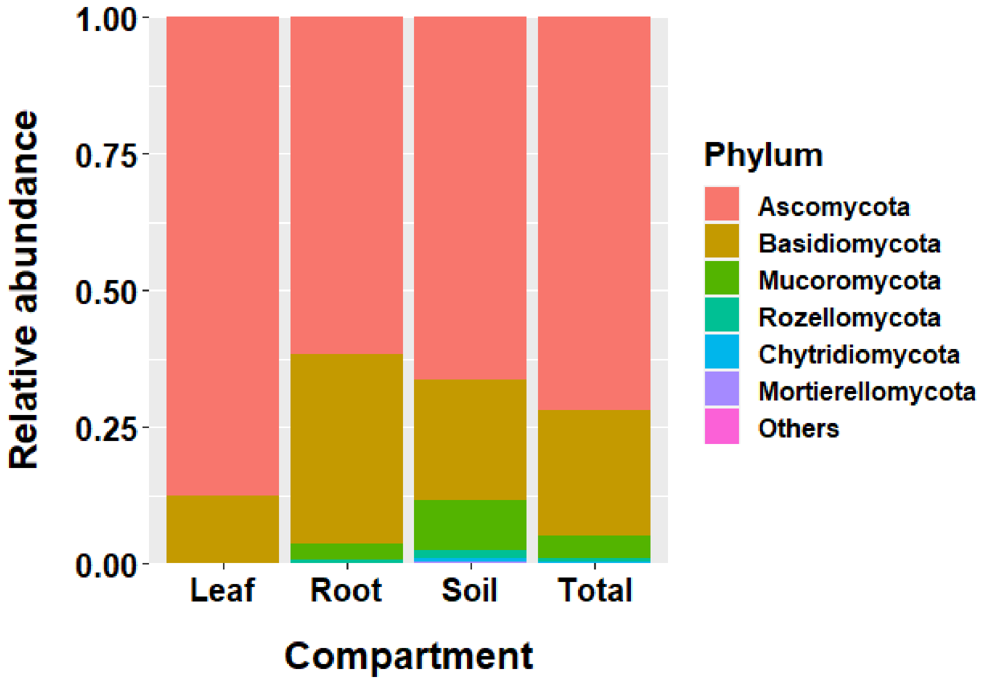

FIG S1 The distribution of fungal phyla among total dataset and sub-datasets of three compartments of leaf, root and soil.

Fig. S2

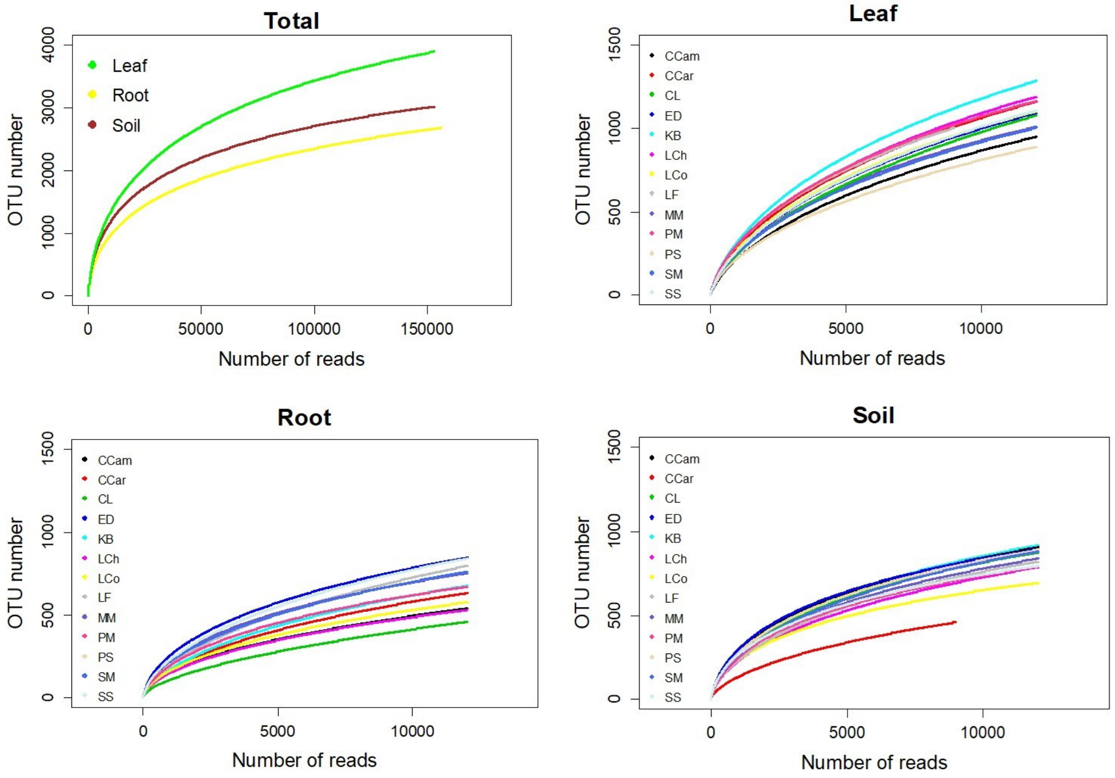

FIG S2 Rarefaction curves for observed fungal OTUs recovered from three compartments (A) of leaf (B) and root (C) and soil (D) samples among 13 woody plant species in subtropical forest. Plant species names: CCam, *Cinnamomum camphora*, CCar, *Castanopsis carlesii*, CL, *Cunninghamia lanceolate*, ED, *Elaeocarpus decipiens*, KB, *Koelreuteria bipinnata*, LCh, *Liriodendron chinense*, LCo, *Lindera communis*, LF, *Liquidambar formosana*, MM, *Michelia macclurei*, PM, *Pinus massoniana*, PS, *Photinia serrulate*, SM, *Sapindus mukorossi*, SS, *Schima superba*.

Fig. S3

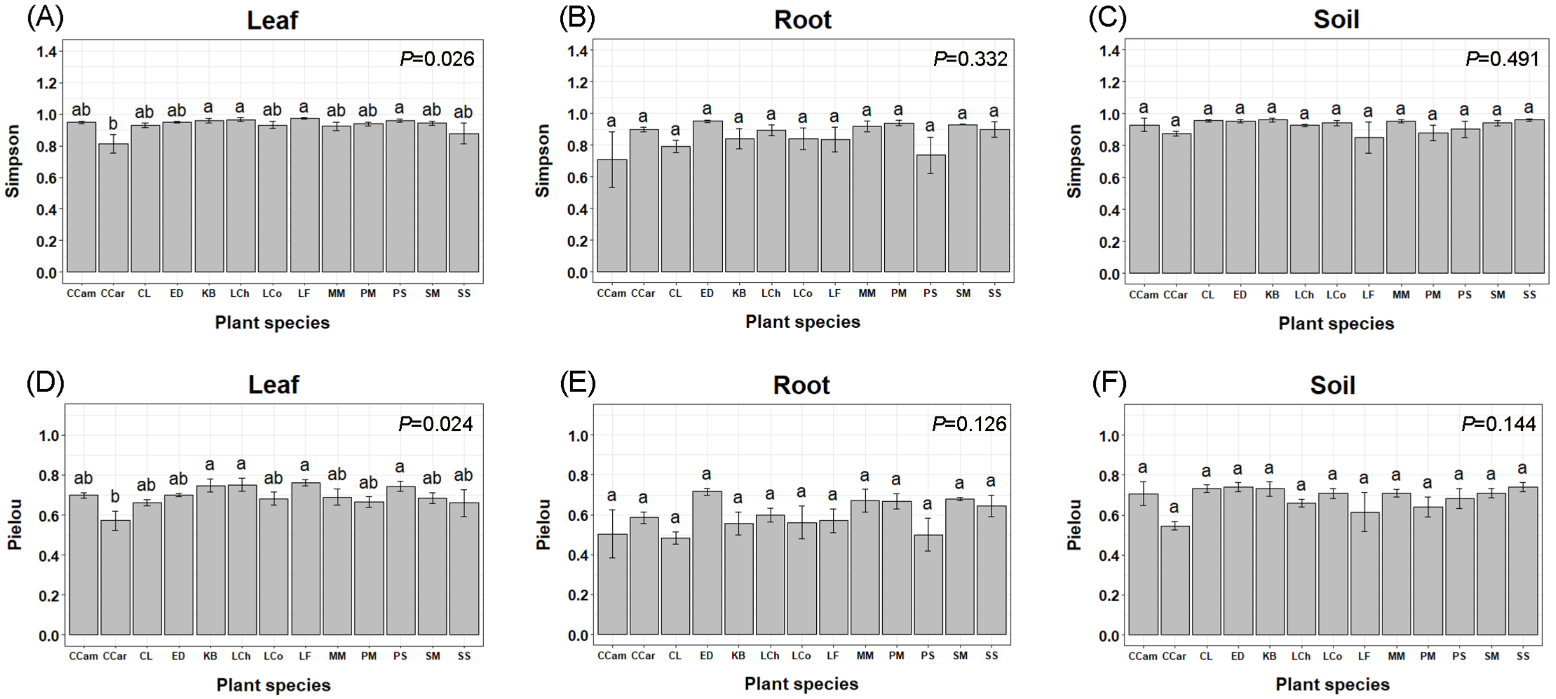

FIG S3 Simpsons index comparison amongst 13 plant species for each of three compartments of leaf (A), root (B) and soil (C), and Pielou index comparison amongst 13 plant species for each of three compartments of leaf (D), root (E) and soil (F). Plant species names: CCam, *Cinnamomum camphora*, CCar, *Castanopsis carlesii*, CL, *Cunninghamia lanceolate*, ED, *Elaeocarpus decipiens*, KB, *Koelreuteria bipinnata*, LCh, *Liriodendron chinense*, LCo, *Lindera communis*, LF, *Liquidambar formosana*, MM, *Michelia macclurei*, PM, *Pinus massoniana*, PS, *Photinia serrulate*, SM, *Sapindus mukorossi*, SS, *Schima superba*.

Fig. S4

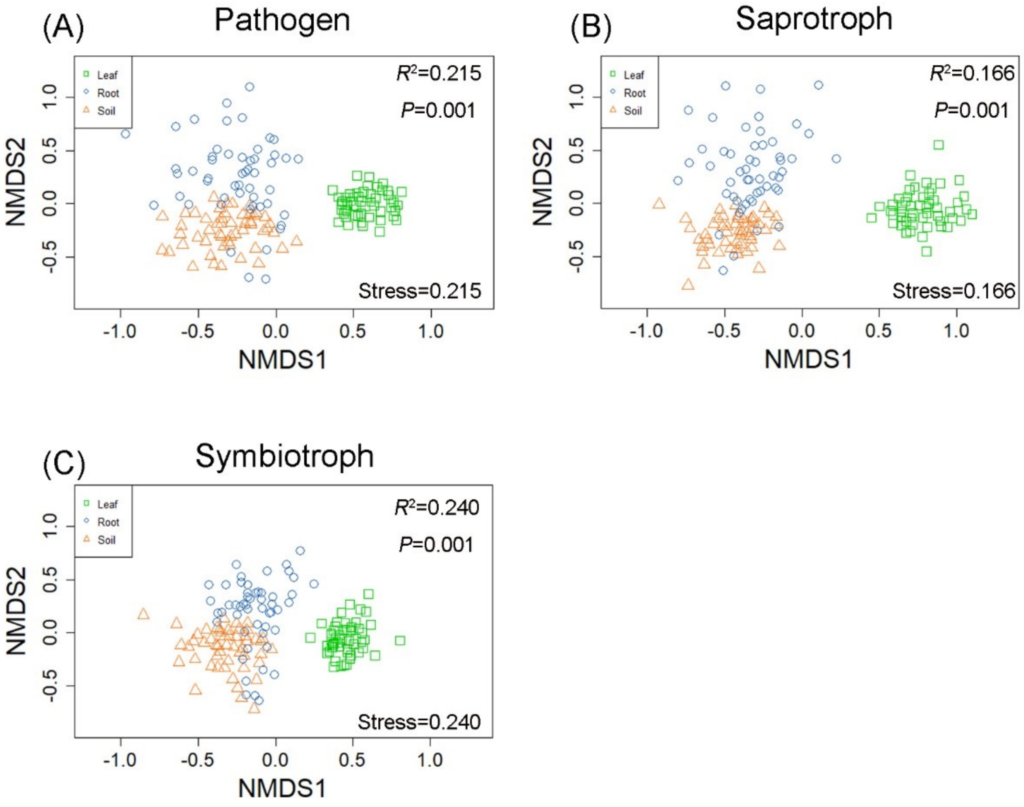

FIG S4 Non-metric multidimensional scaling (NMDS) of the fungal community compositions for pathogen (A) and saprotroph (B), and symbiotroph (C) fungi. R^2 and P values represent the result of PERMANOVA analysis for each fungal functional guild.

Fig. S5

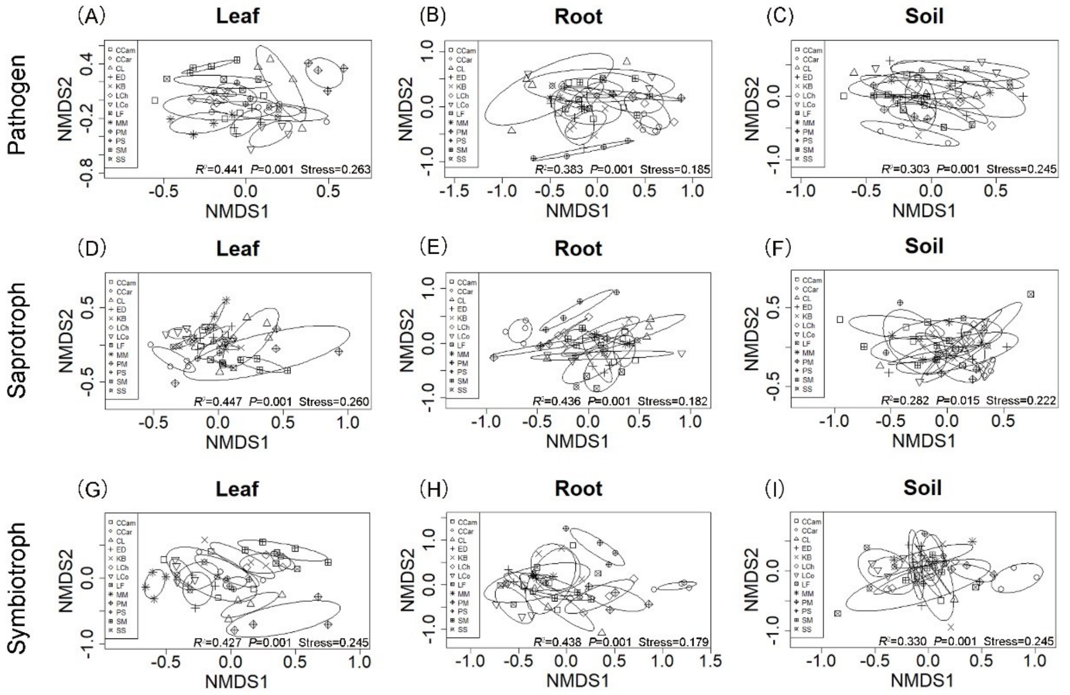

FIG S5 Non-metric multidimensional scaling (NMDS) of the fungal community compositions in 13 plant species within each of three compartments of leaf and root, and soil in pathogen and saprotroph, and symbiosis fungi. (A), (B) and (C) represent pathogenic fungal community compositions of leaf, root and soil, respectively. Similarly, (D), (E) and (F) represent saprotroph fungal community compositions of leaf, root and soil, respectively, and (G), (H) and (I) represent symbiotroph fungal community compositions of leaf, root and soil, respectively. Ellipses indicate 95% confidence intervals around centroids of different plants. R^2 and P values represent the results of PERMANOVA analyses. Plant species names: CCam, *Cinnamomum camphora*, CCar, *Castanopsis carlesii*, CL, *Cunninghamia lanceolate*, ED, *Elaeocarpus decipiens*, KB, *Koelreuteria bipinnata*, LCh, *Liriodendron chinense*, LCo, *Lindera communis*, LF, *Liquidambar formosana*, MM, *Michelia macclurei*, PM, *Pinus massoniana*, PS, *Photinia serrulate*, SM, *Sapindus mukorossi*, SS, *Schima superba*.

Fig. S6

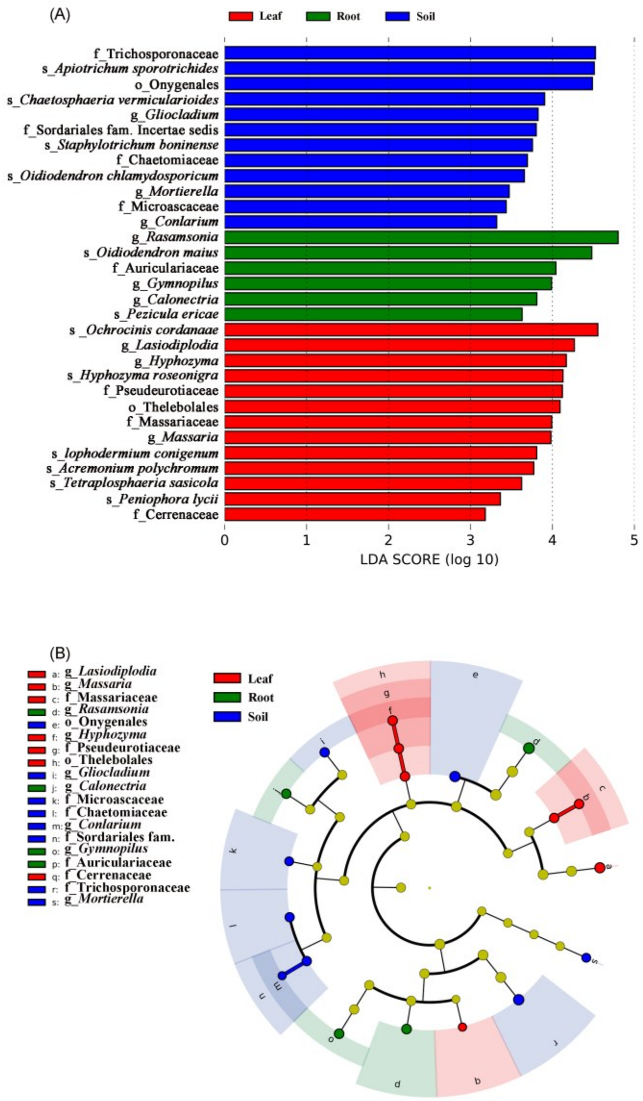

FIG S6 (A) Linear discriminant analysis (LDA) effect size (LEfSe) showing significant differences in the relative abundances of fungal taxa across leaf (red, 13), root (green, six) and soil (blue, 12), with a logarithmic LDA score of 3.0 as the threshold. (B) LEfSe cladogram indicating the phylogenetic distribution of 19 fungal lineages (above species level). The size of each small circle is roughly proportional to the relative abundance of that given taxa. Six rings of the cladogram represent phylogenetic levels from domain to genus from the inside outwards. The small yellow circles represent the fungal taxa with no significant differences, while small circles and sectors with other different colors represent the significantly enriched fungal taxa in different compartments. Biological classifications are listed beside the cladogram, ‘o’ refers to order, ‘f’ refers to family, ‘g’ refers to genus and ‘s’ refers to species.

TABLE S1

Summary of ANOVA results of the effects of compartments and plant identity (species, leaf traits, mycorrhizal type, and mean leaf area) on the alpha-diversities (richness, Shannon-Wiener and Simpson's indices, and Pielou's evenness) based on total, leaf, root, and soil datasets, respectively

**Dataset**	**Df**	**Richness**		**Shannon**		**Simpson**		**Pielou**	
		***F* value**	***P* value**	***F* value**	***P* value**	***F* value**	***P* value**	***F* value**	***P* value**
**Total**									
compartment	2	110.400	**<0.001**	26.560	**<0.001**	7.981	**<0.001**	15.470	**<0.001**
plant species	12	3.220	**<0.001**	2.138	**0.020**	1.073	0.389	1.785	0.059
compartment × species	24	3.028	**<0.001**	1.938	**0.011**	1.337	0.157	1.678	**0.037**
leaf traits	2	1.566	0.212	0.910	0.405	0.443	0.643	0.562	0.571
mycorrhizal type	1	5.057	**0.026**	5.414	**0.021**	0.483	0.488	4.167	**0.043**
mean leaf area	1	0.628	0.429	0.303	0.583	0.008	0.927	0.157	0.692
**Leaf**									
plant species	12	3.026	**0.005**	2.378	**0.021**	2.290	**0.026**	2.317	**0.024**
leaf traits	2	5.181	**0.009**	4.311	**0.019**	2.587	0.086	3.837	**0.029**
mycorrhizal type	1	12.420	**0.001**	12.140	**0.001**	7.777	**0.008**	10.740	**0.002**
mean leaf area	1	6.220	**0.016**	9.637	**0.003**	3.057	0.087	9.293	**0.004**
**Root**									
plant species	12	4.550	**<0.001**	2.057	**0.045**	1.178	0.332	1.620	0.126
leaf traits	2	2.703	0.077	0.373	0.690	0.142	0.868	0.108	0.898
mycorrhizal type	1	0.173	0.679	0.317	0.576	1.610	0.210	0.593	0.445
mean leaf area	1	0.006	0.937	0.029	0.866	0.086	0.771	0.031	0.862
**Soil**									
plant species	12	2.003	0.052	1.680	0.111	0.972	0.491	1.565	0.144
leaf traits	2	0.572	0.568	0.048	0.953	0.216	0.806	0.169	0.845
mycorrhizal type	1	7.127	**0.010**	10.030	**0.003**	3.885	0.054	8.957	**0.004**
mean leaf area	1	0.021	0.887	0.702	0.406	0.903	0.347	1.133	0.292

TABLE S2

Summary of the PERMANOVA analyses for the fungal community compositions based on total, leaf, root, and soil datasets, respectively

**Dataset**	**PERMANOVA**		
effect origin	Df	R^2	*P* value
**Total**			
compartment	2	0.180	**0.001**
plant species	12	0.138	**0.001**
compartment × species	38	0.518	**0.001**
leaf traits	2	0.029	**0.001**
mycorrhizal type	1	0.023	**0.001**
mean leaf area	1	0.012	**0.006**
**Leaf**			
plant species	12	0.511	**0.001**
leaf traits	2	0.146	**0.001**
mycorrhizal type	1	0.059	**0.001**
mean leaf area	1	0.056	**0.001**
**Root**			
plant species	12	0.421	**0.001**
leaf traits	2	0.074	**0.001**
mycorrhizal type	1	0.060	**0.001**
mean leaf area	1	0.031	**0.009**
**Soil**			
plant species	12	0.305	**0.001**
leaf traits	2	0.053	**0.006**
mycorrhizal type	1	0.061	**0.001**
mean leaf area	1	0.027	**0.047**

TABLE S3

Summary of the beta dispersion analyses for the fungal community compositions based on total, leaf, root, and soil datasets, respectively

**Dataset**	**Df**	***F* value**	***P* value**
**Compartment effect**			
Total	2	31.63	< 0.001
**Species effect**			
Leaf	12	1.633	0.123
Root	12	0.551	0.867
Soil	12	0.543	0.872
